# An SIR epidemic model for COVID-19 spread with fuzzy parameter: the case of Indonesia

**DOI:** 10.1186/s13662-021-03263-6

**Published:** 2021-02-11

**Authors:** Muhammad Abdy, Syafruddin Side, Suwardi Annas, Wahyuddin Nur, Wahidah Sanusi

**Affiliations:** 1 Department of Mathematics, Faculty of Mathematics and Natural Science, Universitas Negeri Makassar, Makassar, Indonesia; 2 Department of Statistics, Faculty of Mathematics and Natural Science, Universitas Negeri Makassar, Makassar, Indonesia; 3 Department of Mathematics, Faculty of Mathematics and Natural Science, Universitas Sulawesi Barat, Majene, Indonesia

**Keywords:** SIR model, Fuzzy parameter, COVID-19, Vaccination, Treatment, Health protocol

## Abstract

The aim of this research is to construct an SIR model for COVID-19 with fuzzy parameters. The SIR model is constructed by considering the factors of vaccination, treatment, obedience in implementing health protocols, and the corona virus-load. Parameters of the infection rate, recovery rate, and death rate due to COVID-19 are constructed as a fuzzy number, and their membership functions are used in the model as fuzzy parameters. The model analysis uses the generation matrix method to obtain the basic reproduction number and the stability of the model’s equilibrium points. Simulation results show that differences in corona virus-loads will also cause differences in the transmission of COVID-19. Likewise, the factors of vaccination and obedience in implementing health protocols have the same effect in slowing or stopping the transmission of COVID-19 in Indonesia.

## Introduction

A new virus that can cause an increase in pneumonia first appeared in Wuhan, China, at the beginning of December 2019. The virus was called SARS-COV-2 and the disease associated with the virus was called COVID-19 [1]. The rapid spread of the disease throughout the world has made the World Health Organization declare the COVID-19 outbreak a global pandemic on March 12, 2020 [2]. COVID-19 is transmitted by a person infected with COVID-19 through physical contact with another person, or through small droplets from the mouth of a person with COVID-19 that is touched by another person. According to the data collected by John Hopkins University, as of the beginning of November 2020, COVID-19 has spread to 219 countries in the world with the total number of cases infected with COVID-19 reaching 53,699,160, with 1,308,261 deaths, and 37,469,072 people have been declared cured.

The spread of COVID-19 to Indonesia has been evident since the first confirmed case on March 2, 2020. COVID-19 is continuing to spread to all provinces, and as of the beginning of November 2020 it has reached a total of 457,735 infected cases, 15,037 deaths, and 385,094 people have been declared cured [3].

A number of mathematicians have performed various studies to make a model and predict the spread of COVID-19 since it is becoming a pandemic. The first study [4] to predict COVID-19 in China was conducted using GLM method and Richard’s model. Then [5] predicted COVID-19 in Indonesia based on early endemic data using Richard’s curve. Other models and predictions using statistical approaches [6–10] or those using SIR, SEIR, and their extensions [11–16] have been widely performed. Moreover, some researchers used fractional order in epidemic models to model the spread of COVID-19. Fractional order derivative with Mittag-Leffler function as a nonsingular kernel type [17] and Caputo derivative [18–20] are used in modeling the transmission of COVID-19. Then [21, 22] considered the fractal-fractional derivative in the Atangana–Baleanu sense to obtain the stability of the model, and [23] presented the existence and uniqueness solution of the model via fractal-fractional operators. However, the parameters used in any existing SIR epidemic model and its extensions employ crisp numbers, whereas uncertainty in parameters and heterogeneity in the population are very possible to occur. Therefore the use of uncertain parameters or fuzzy parameters is very important, because the model will reflect the real world problems. Supporting studies could be used as references, such as the fuzzy epidemic models for human infectious diseases [24–26], other models considering the uncertainty of parameter space and heterogeneity of a population [27, 28], fuzzy dynamic systems [29], and dynamic behavior of an epidemic model with fuzzy transmission [30].

In this study, we consider a mathematical model of SIR in a normalized form with three control parameters, namely vaccination control, treatment control and implementation of health protocols. The parameters of infection rate, recovery rate, and death rate due to COVID-19 are treated as fuzzy numbers that depend on individual virus-load.

## Method

The method used to construct the model is the SIR model by considering vaccination, treatment, and the implementation of health protocols as control parameters. The parameters of the infection rate, recovery rate, and death rate due to COVID-19 are constructed as a membership function of a fuzzy number [28]. These parameters depend on the corona virus-load in an individual and control parameters. The model analysis uses the generation matrix method to obtain the basic reproduction number and stability of the SIR model for the spread of COVID-19. The numerical simulation of the model uses data on the number of COVID-19 cases in Indonesia [3]. The parameters of vaccination effectiveness, treatment effectiveness, the level of obedience in implementing health protocols, and corona virus-load are assumed in this simulation.

## Results and discussion

### SIR model of COVID-19

**Figure 1:**
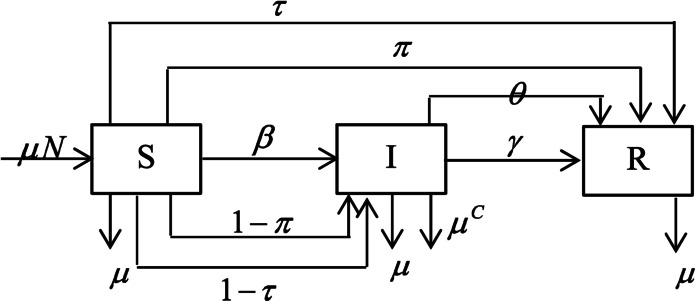
The schematic of the model SIR of COVID-19 spread transmission flow

Consider an SIR model for COVID-19 that describes the dynamics of direct transmission of COVID-19 with interactions between suspected and infected, change from being infected to recovering, pure birth/death rates, vaccine effectiveness, treatment effectiveness, obedience in implementing health protocols, and deaths due to the COVID-19 infection. The schematic diagram of the COVID-19 transmission flow is given in Fig. 1, and the model is mathematically described as follows:

1 $$\begin{aligned}& \frac{dS}{dt} = \mu - \beta (1 - \tau ) (1 - \pi )SI - (\mu + \tau + \pi )S, \end{aligned}$$

2 $$\begin{aligned}& \frac{dI}{dt} = \beta (1 - \tau ) (1 - \pi )SI - \bigl(\mu + \mu ^{C} + \theta + \gamma \bigr)I, \end{aligned}$$

3 $$\begin{aligned}& \frac{dR}{dt} = (\theta + \gamma )I + (\pi + \tau )S - \mu R, \end{aligned}$$

where *S* is the proportion of susceptible individuals, *I* is the proportion of infected individuals, *R* is the proportion of recovered individuals, *β* is the infection rate parameter; *γ* is the recovery rate parameter; *μ* is the natural birth/death rate parameter, *τ* is the vaccine effectiveness parameter, *θ* is the treatment effectiveness parameter, *π* is the effectiveness of obedience in implementing health protocols, $\mu ^{C}$ is the death rate parameter due to COVID-19. Now, we can extend the SIR model (1)–(3) by considering the heterogeneity of the corona virus-load in each individual, where individuals with different amount of the corona virus-load contribute differently in transmitting COVID-19.

### The SIR fuzzy model of COVID-19 spread

Consider the SIR model for COVID-19 in (1)–(3). Let Ω be the corona virus-load in an individual. Now, we consider the heterogeneity in the model by considering the power to infect in each individual as a function of the corona virus-load Ω. Therefore, the higher the corona virus-load in an individual, the higher the chance of the corona virus transmission in a contact interaction. By considering the corona virus-load Ω in each individual, the parameters *β*, $\mu ^{C}$, and *γ* can be viewed as a function of the corona virus-load Ω. Thus, model (1)–(3) can be extended to a model, which we hereinafter call the fuzzy SIR model, represented as follows:

4 $$\begin{aligned}& \frac{dS}{dt} = \mu - \beta (\Omega ) (1 - \tau ) (1 - \pi )SI - (\mu + \tau + \pi )S, \end{aligned}$$

5 $$\begin{aligned}& \frac{dI}{dt} = \beta (\Omega ) (1 - \tau ) (1 - \pi )SI - \bigl(\mu + \mu ^{C}(\Omega ) + \theta + \gamma (\Omega )\bigr)I, \end{aligned}$$

6 $$\begin{aligned}& \frac{dR}{dt} = \bigl(\theta + \gamma (\Omega )\bigr)I + (\pi + \tau )S - \mu R. \end{aligned}$$

Let $\beta = \beta (\Omega )$ be the chance of transmission between a suspected and an infected individual with the amount of the corona virus-load Ω. Some values of *β* are more reasonable compared to some others, and it turns *β* into a membership function of fuzzy numbers. To construct the membership function, we assume that if the number of corona virus-loads in an individual is relatively low, the chance of transmission is negligible, and there is a minimum corona virus-load $\Omega _{\min } $ needed to be able to transmit to other individuals. Furthermore, there is a certain amount of corona virus-load $\Omega _{0}$, where the transmission rate is maximum and equal to one. However, we assume that the total amount of corona virus-load Ω on a person is limited by $\Omega _{\max } $ [28]. We can also consider that the vaccination and the discipline to follow health protocols will affect the infection rate of COVID-19. Let *τ* and *π* be the parameters representing the vaccine effectiveness and the level of discipline in implementing health protocols, respectively. Then fuzzy membership function of the infectivity contact rate is given as follows:

7 $$ \beta (\Omega ) = \textstyle\begin{cases} 0&\text{if } \Omega \le \Omega _{\min }, \\ \frac{(\Omega - \Omega _{\min } )(1 - \tau )(1 - \pi )}{\Omega _{0} - \Omega _{\min }}& \text{if } \Omega _{\min } < \Omega < \Omega _{0}, \\ (1 - \tau )(1 - \pi )&\text{if } \Omega _{0} \le \Omega < \Omega _{\max }. \end{cases} $$

**Figure 2:**
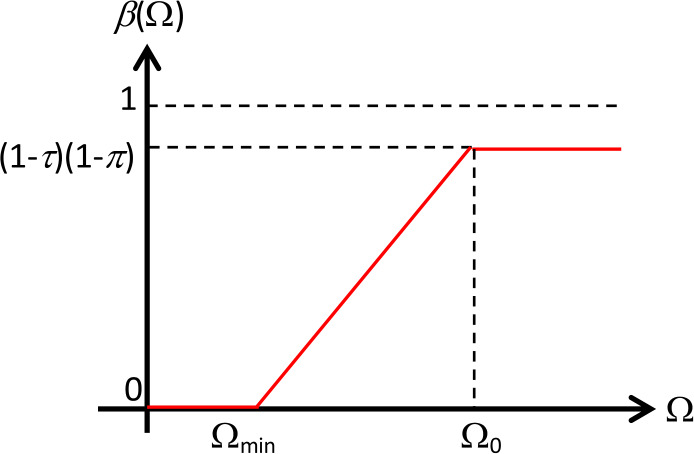
The graph of membership function of *β*

The graphic of $\beta (\Omega )$ is given in Fig. 2.

**Figure 3:**
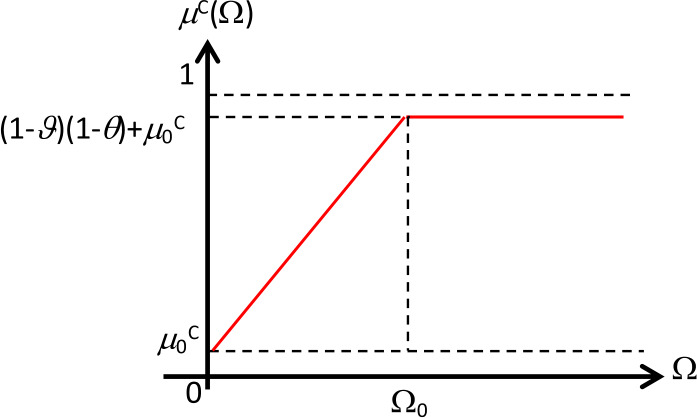
The graph of the membership function of $\mu ^{C}$

The death rate due to COVID-19 infection can also be assumed as a membership function of a fuzzy number. The function is an increasing function of corona virus-load Ω. However, due to some reasons, such as a person infected with COVID-19 suffering from other diseases, immunity power, availability of medicine, etc., the function might not reach its maximum value equal to one. Likewise, treatment for COVID-19 will affect the death rate due to COVID-19 infection. Therefore, we assume that the maximum value of the function $\mu ^{C}(\Omega )$ is $(1 - \vartheta )(1 - \theta ) + \mu _{0}^{C}\theta $, with ($0 \le \vartheta \le 1$); ($0 \le \theta \le 1$). Thus, we can define the function $\mu ^{C}(\Omega )$ as follows (depicted in Fig. 3):

8 $$ \mu ^{C}(\Omega ) = \textstyle\begin{cases} ((1 - \vartheta ) - \mu _{0}^{C})(1 - \theta )\frac{\Omega }{\Omega _{0}} + \mu _{0}^{C} & \text{if } 0 \le \Omega < \Omega _{0}, \\ (1 - \vartheta )(1 - \theta ) + \theta \mu _{0}^{C} & \text{if } \Omega _{0} \le \Omega, \end{cases} $$

where $\mu _{0}^{C}$; ($0 < \mu _{0}^{C} < 1$) is the lowest death rate due to COVID-19 infection and *θ* is the treatment effectiveness.

**Figure 4:**
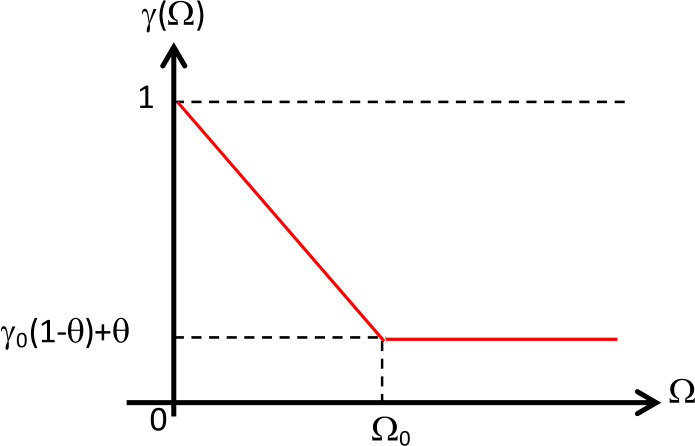
The graph of the membership function of *γ*

The recovery rate of the COVID-19 infection group $\gamma = \gamma (\Omega )$ is also a function of the corona virus-load Ω. The higher the corona virus-load Ω, the longer the recovery process will take from infection. So, $\gamma (\Omega )$ is a decreasing function. Moreover, we can also consider the effect of the treatment on the rate of recovery. Thus the fuzzy membership function can be defined as follows (depicted in Fig. 4):

9 $$ \gamma (\Omega ) = \textstyle\begin{cases} (\gamma _{0} - 1)(1 - \theta )\frac{\Omega }{\Omega _{0}} + 1 & \text{if } 0 \le \Omega < \Omega _{0}, \\ \gamma _{0}(1 - \theta ) + \theta & \text{if } \Omega \ge \Omega _{0}, \end{cases} $$

where $\gamma _{0}$ is the lowest recovery rate.

In the fuzzy SIR model, the membership function of the infection rate $\beta (\Omega )$, the recovery rate $\gamma (\Omega )$, and the death rate $\mu ^{C}(\Omega )$ due to COVID-19 infection are treated as fuzzy parameters of the model.

### The equilibrium points and the basic reproduction number

There are two equilibrium points in model (4)–(6), namely the disease-free equilibrium point and the endemic equilibrium point. To determine these two equilibrium points, each of the equations in the equations must be equal to zero, that is, $\frac{dS}{dt} = 0$, $\frac{dI}{dt} = 0$, $\operatorname{dan} \frac{dR}{dt} = 0$ so that:

10 $$\begin{aligned}& \mu - \beta (\Omega ) (1 - \tau ) (1 - \pi )SI - (\mu + \tau + \pi )S = 0, \end{aligned}$$

11 $$\begin{aligned}& \beta (\Omega ) (1 - \tau ) (1 - \pi )SI - \bigl(\mu + \mu ^{C}( \Omega ) + \theta + \gamma (\Omega )\bigr)I = 0, \end{aligned}$$

12 $$\begin{aligned}& \bigl(\theta + \gamma (\Omega )\bigr)I + (\pi + \tau )S - \mu R = 0, \end{aligned}$$

then the equilibrium points for *S*, *I*, and *R* are as follows.

#### The disease-free equilibrium point for the SIR fuzzy model

The points of equilibrium for disease free are conditions where there is no spread of COVID-19, namely $I = I^{0} = 0$. Thus, from Eq. (4), we obtain

13 $$ S = S^{0} = \frac{\mu }{\pi + \tau + \mu }, $$

from Eq. (6) and Eq. (13), we obtain

14 $$ R = R^{0} = \frac{\pi + \tau }{\pi + \tau + \mu }. $$

Thus, the disease-free equilibrium point for the SIR fuzzy model (4)–(6) is

15 $$ E^{0} = \bigl(S^{0}, I^{0}, R^{0}\bigr) = \biggl( \frac{\mu }{\pi + \tau + \mu }, 0, \frac{\pi + \tau }{\pi + \tau + \mu } \biggr). $$

#### The endemic equilibrium point for the SIR fuzzy model

Endemic equilibrium points are conditions where there is the possibility of disease spread, namely $S = S^{*} \neq 0$, $I = I^{*} \neq 0$, and $R = R^{*} \neq 0$. Thus, from Eqs. (4)–(6), we obtain the endemic equilibrium points for the SIR fuzzy model as follows:

16 $$\begin{aligned}& S^{*} = \frac{\theta + \mu ^{C}(\Omega ) + \gamma (\Omega ) + \mu }{\beta (\Omega )(1 - \pi )(1 - \tau )}, \end{aligned}$$

17 $$\begin{aligned}& I^{*} = \frac{\mu }{\theta + \mu ^{C}(\Omega ) + \gamma (\Omega ) + \mu } - \frac{\pi + \tau + \mu }{\beta (\Omega )(1 - \tau )(1 - \pi )}, \end{aligned}$$

18 $$\begin{aligned}& R^{*} = \frac{(\theta + \gamma (\Omega )) I^{*} + (\pi + \tau )S^{*}}{\mu }. \end{aligned}$$

Thus,

19 $$ E^{1} = \bigl(S^{*}, I^{*}, R^{*}\bigr) = \begin{pmatrix} \frac{\theta + \mu ^{C}(\Omega ) + \gamma (\Omega ) + \mu }{\beta (1 - \pi )(1 - \tau )}, \\ \frac{\mu }{\theta + \mu ^{C}(\Omega ) + \gamma (\Omega ) + \mu } - \frac{\pi + \tau + \mu }{\beta (\Omega )(1 - \tau )(1 - \pi )}, \\ \frac{(\theta + \gamma (\Omega )) I^{*} + (\pi + \tau )S^{*}}{\mu } \end{pmatrix}. $$

The basic reproductive number $\Re _{0}$ for system (1)–(3) is determined using the next generation matrix method [31]. Based on Eqs. (1)–(3), to determine $\Re _{0}$:

Let $F = \beta (1 - \tau )(1 - \pi )SI$ and $V = (\mu + \mu ^{C} + \theta + \gamma )I$, then we obtain

$$ F' = \beta (1 - \tau ) (1 - \pi )S, \qquad V' = \mu + \mu ^{C} + \theta + \gamma , \quad \mbox{and} \quad \bigl(V'\bigr)^{ - 1} = \frac{1}{\mu + \mu ^{C} + \theta + \gamma }. $$

The dominant eigenvalue of $F'(V')^{ - 1}$ represents $\Re _{0} = \rho (F'(V')^{ - 1})$, which is

20 $$ \Re _{0} = \frac{\beta \mu (1 - \tau )(1 - \pi )}{(\pi + \tau + \mu )(\theta + \mu ^{C} + \gamma + \mu )}. $$

As in this case, we have taken $\beta = \beta (\Omega )$, $\mu ^{C} = \mu ^{C}(\Omega )$, and $\gamma = \gamma (\Omega )$, then we write

21 $$ \Re _{0}(\Omega ) = \frac{\beta (\Omega )\mu (1 - \tau )(1 - \pi )}{(\pi + \tau + \mu )(\theta + \mu ^{C}(\Omega ) + \gamma (\Omega ) + \mu )}, $$

$\Re _{0}(\Omega )$ is the basic reproductive number which is a function of virus-load Ω. $\beta (\Omega )$, $\mu ^{C}(\Omega )$, and $\gamma (\Omega )$ are fuzzy parameters which are a function of the virus-load Ω.

### Stability analysis

#### Theorem 1

*If* $\Re _{0}(\Omega ) < 1$, *then the disease*-*free equilibrium point for system* (4)*–*(6) *is locally asymptotically stable*, *and if* $\Re _{0}(\Omega ) > 1$, *then the disease*-*free equilibrium point of the system is unstable*.

#### Proof

From equation system (4)–(6), we write the Jacobian matrix *J* as follows:

$$ J = \begin{bmatrix} - \beta (\Omega )(1 - \tau )(1 - \pi )I - (\pi + \tau + \mu ) & - \beta (\Omega )(1 - \tau )(1 - \pi )S & 0 \\ \beta (\Omega )(1 - \tau )(1 - \pi )I & \beta (\Omega )(1 - \tau )(1 - \pi )S - (\theta + \mu ^{C}(\Omega ) + \gamma (\Omega ) + \mu ) & 0 \\ 0 & 0 & - \mu \end{bmatrix}. $$

Substituting the value $I = 0$ and $S = \frac{\mu }{\pi + \tau + \mu } $, we obtain matrix $J_{0}$

$$\begin{aligned}& J_{0} = \begin{bmatrix} - (\pi + \tau + \mu ) & - \beta (\Omega )(1 - \tau )(1 - \pi )\frac{\mu }{\pi + \tau + \mu } & 0 \\ 0 & \beta (\Omega )(1 - \tau )(1 - \pi )S - (\theta + \mu ^{C}(\Omega ) + \gamma (\Omega ) + \mu ) & 0 \\ 0 & 0 & - \mu \end{bmatrix}, \\& \operatorname{eigen}(J_{0}) \\& \quad = (\lambda + \pi + \tau + \mu ) (\lambda + \mu ) \biggl( \bigl(\lambda + \theta + \mu ^{C}(\Omega ) + \gamma (\Omega ) + \mu \bigr) - \frac{\beta (\Omega )\mu (1 - \tau )(1 - \pi )}{\pi + \tau + \mu } \biggr), \end{aligned}$$

we obtain

$$\begin{aligned}& \lambda _{1} = - (\pi + \tau + \mu ), \\& \lambda _{2} = - \mu , \\& \lambda _{3} = - \biggl( - \frac{\beta (\Omega )\mu (1 - \tau )(1 - \pi )}{(\pi + \tau + \mu )} + \bigl(\theta + \mu ^{C}(\Omega ) + \gamma (\Omega ) + \mu \bigr) \biggr) \\& \hphantom{\lambda _{3}}= - \bigl(\theta + \mu ^{C}(\Omega ) + \gamma ( \Omega ) + \mu \bigr) \biggl( - \frac{\beta (\Omega )\mu (1 - \tau )(1 - \pi )}{(\pi + \tau + \mu )(\theta + \mu ^{C}(\Omega ) + \gamma (\Omega ) + \mu )} + 1 \biggr) \\& \hphantom{\lambda _{3}}= - \bigl(\theta + \mu ^{C}(\Omega ) + \gamma ( \Omega ) + \mu \bigr) \bigl( - \Re _{0}(\Omega ) + 1 \bigr). \end{aligned}$$

If $\Re _{0}(\Omega ) < 1$, then $\lambda _{3} < 0$, and if $\Re _{0}(\Omega ) > 1$, then $\lambda _{3} > 0$. □

#### Theorem 2

*If* $\Re _{0}(\Omega ) > 1$, *then the endemic equilibrium point of system* (4)*–*(6) *is locally asymptotically stable*.

#### Proof

From equation system (4)–(6) and the endemic equilibrium point, we obtain Jacobian matrix $J_{1}$ as follows:

$$ J_{1} = \begin{bmatrix} - \beta ( \Omega ) ( 1 - \tau ) ( 1 - \pi )I^{*} - ( \pi + \tau + \mu ) & - \beta ( \Omega ) ( 1 - \tau ) ( 1 - \pi )S^{*} & 0 \\ \beta ( \Omega ) ( 1 - \tau ) ( 1 - \pi )I^{*} & \beta ( \Omega ) ( 1 - \tau ) ( 1 - \pi )S^{*} - ( \theta + \mu ^{c} ( \Omega ) + \gamma ( \Omega ) + \mu ) & 0 \\ 0 & 0 & - \mu \end{bmatrix}. $$

We assume that $j_{1} = \beta ( \Omega ) ( 1 - \tau ) ( 1 - \pi )I^{*} + ( \pi + \tau + \mu )$, $j_{2} = \beta ( \Omega ) ( 1 - \tau ) ( 1 - \pi )S^{*}$, $j_{3} = \beta ( \Omega ) ( 1 - \tau ) ( 1 - \pi )I^{*}$, and $j_{4} = \beta ( \Omega ) ( 1 - \tau ) ( 1 - \pi )S^{*} - ( \theta + \mu ^{c} ( \Omega ) + \gamma ( \Omega ) + \mu )$. Thus,

$$ J_{1} = \begin{bmatrix} - j_{1} & - j_{2} & 0 \\ j_{3} & j_{4} & 0 \\ 0 & 0 & - \mu \end{bmatrix}. $$

The eigenvalues of $J_{1}$ are roots of $P_{1} ( \lambda )$:

$$\begin{aligned} P_{1} ( \lambda ) &= ( \lambda + \mu ) \bigl[ ( \lambda + j_{1} ) ( \lambda - j_{4} ) + j_{2}j_{3} \bigr] \\ &= ( \lambda + \mu ) \bigl[ \lambda ^{2} + ( j_{1} - j_{4} )\lambda - j_{1}j_{4} + j_{2}j_{3} \bigr] = ( \lambda + \mu )P_{2} ( \lambda ). \end{aligned}$$

It is easy to see that $\lambda _{1} = - \mu $ is one of the eigenvalues $P_{1} ( \lambda )$. The other eigenvalues are the solutions of $P_{2} ( \lambda ) = 0$. Based on the Routh–Hurwitz condition, $P_{2} ( \lambda )$ has two roots which have a negative real part if $j_{1} - j_{4} > 0$ and $j_{2}j_{3} - j_{1}j_{4} > 0$.

$$\begin{aligned} j_{1} - j_{4} =& \bigl[ \beta ( \Omega ) ( 1 - \tau ) ( 1 - \pi )I^{*} + ( \pi + \tau + \mu ) \bigr] \\ &{}- \bigl[ \beta ( \Omega ) ( 1 - \tau ) ( 1 - \pi )S^{*} - \bigl( \theta + \mu ^{c} ( \Omega ) + \gamma ( \Omega ) + \mu \bigr) \bigr] \\ =& \bigl[ \bigl( \Re _{0} ( \Omega ) - 1 \bigr) ( \pi + \tau + \mu ) + ( \pi + \tau + \mu ) \bigr] \\ &{}- \biggl[ \beta ( \Omega ) ( 1 - \tau ) ( 1 - \pi ) \biggl( \frac{\mu - ( \theta + \mu ^{c} ( \Omega ) + \gamma ( \Omega ) + \mu )\frac{ ( \Re _{0} ( \Omega ) - 1 ) ( \pi + \tau + \mu )}{\beta ( \Omega ) ( 1 - \tau ) ( 1 - \pi )}}{\pi + \tau + \mu } \biggr) \\ &{}- \bigl( \theta + \mu ^{c} ( \Omega ) + \gamma ( \Omega ) + \mu \bigr) \biggr] \\ =& \Re _{0} ( \Omega ) ( \pi + \tau + \mu ) + \bigl( 1 - \Re _{0} ( \Omega ) \bigr) \bigl( \theta + \mu ^{c} ( \Omega ) + \gamma ( \Omega ) + \mu \bigr) \\ &{}+ \bigl( \theta + \mu ^{c} ( \Omega ) + \gamma ( \Omega ) + \mu \bigr) \bigl( \Re _{0} ( \Omega ) - 1 \bigr) \\ =& \Re _{0} ( \Omega ) ( \pi + \tau + \mu ). \end{aligned}$$

It is clear that $j_{1} - j_{4} > 0$ if $\Re _{0} ( \Omega ) > 0$.

$$\begin{aligned} j_{2}j_{3} - j_{1}j_{4} =& \bigl[ \bigl( \beta ( \Omega ) ( 1 - \tau ) ( 1 - \pi )S^{*} \bigr) \bigl( \beta ( \Omega ) ( 1 - \tau ) ( 1 - \pi )I^{*} \bigr) \bigr] \\ &{} - \bigl[ \bigl( \beta ( \Omega ) ( 1 - \tau ) ( 1 - \pi )I^{*} + ( \pi + \tau + \mu ) \bigr) \bigl( \beta ( \Omega ) ( 1 - \tau ) ( 1 - \pi )S^{*} \\ &{}- \bigl( \theta + \mu ^{c} ( \Omega ) + \gamma ( \Omega ) + \mu \bigr) \bigr) \bigr] \\ =& \biggl[ \biggl( \frac{\beta ( \Omega ) ( 1 - \tau ) ( 1 - \pi )\mu }{\pi + \tau + \mu } - \bigl( \theta + \mu ^{c} ( \Omega ) + \gamma ( \Omega ) + \mu \bigr) \bigl( \Re _{0} ( \Omega ) - 1 \bigr) \biggr) \\ &{}\times\bigl( \Re _{0} ( \Omega ) - 1 \bigr) ( \pi + \tau + \mu ) \biggr] \\ =& \bigl( \Re _{0} ( \Omega ) - 1 \bigr) ( \pi + \tau + \mu ) \bigl( \theta + \mu ^{c} ( \Omega ) + \gamma ( \Omega ) + \mu \bigr). \end{aligned}$$

It is easy to see that $j_{2}j_{3} - j_{1}j_{4} > 0$ if $\Re _{0} ( \Omega ) > 1$. □

Since the disease-free equilibrium is stable if $\Re _{0}(\Omega ) < 1$ and unstable for $\Re _{0}(\Omega ) > 1$, then system (4)–(6) is at a bifurcation point when $\Re _{0}(\Omega ) = 1$. Let $\Omega ^{*}$ be the bifurcation value of the system, then $\Omega ^{*}$ is the solution of the equation

$$ \beta (\Omega ) (1 - \tau ) (1 - \pi )\mu = (\pi + \tau + \mu ) \bigl(\theta + \mu ^{C}(\Omega ) + \gamma (\Omega ) + \mu \bigr), $$

that is,

$$ \Omega ^{*} = \frac{\mu ((1 - \tau )(1 - \pi ))^{2}\Omega _{0}\Omega _{\min } + \Omega _{0}(\Omega _{0} - \Omega _{\min } )(\pi + \tau + \mu )(\mu _{0}^{C} + 1)}{\mu \Omega _{0} ( (1 - \tau )(1 - \pi ) )^{2} - ( (\pi + \tau + \mu )(\Omega _{0} - \Omega _{\min } ) ) ( (1 - \zeta ) - \mu _{0}^{C})(1 - \theta ) + (\gamma _{0} - 1)(1 - \theta ) )}, $$

where $\Omega ^{*} \le \Omega _{0}$.

In this way, we can think of $\Omega ^{*}$ as a parameter related to the corona virus control in the sense that if a corona virus is transmitted in some number of individuals, it should be noted that Ω is not higher than $\Omega ^{*}$.

#### Corollary

*The disease*-*free equilibrium and the endemic equilibrium of system* (4)*–*(6) *are locally asymptotically stable for* $\Omega < \Omega ^{*}$ *and* $\Omega > \Omega ^{*}$, *respectively*.

### Numerical simulation for transmission of COVID-19 in Indonesia

Numerical simulations are carried out using the initial values for *N*, *S*, *I*, and *R* as in Table 1. The parameters *β*, *γ*, and $\mu ^{C}$ are calculated based on Eqs. (7)–(9) and the parameters in Table 2 and Table 3. The values of $\Omega _{0}$, $\Omega _{\min } $, and Ω should be determined by a virologist, while the value of parameter *ϑ* should be determined by a physician. For each of the controlled parameters, namely the parameters of vaccine effectiveness, level of obedience in implementing health protocols, treatment effectiveness, and corona virus-load, simulations are carried out three times for each parameter with different values, as in Table 3.

**Table 1:** Initial value of the SIR fuzzy model for COVID-19 in Indonesia

Variable	Value	Reference
*N*(0)	269,600,000	[32]
*S*(0)	268,757,171	[3]
*I*(0)	457,735	[3]
*R*(0)	385,094	[3]

**Table 2:** Parameter value of the SIR fuzzy model for COVID-19 in Indonesia

Variable	Value	Reference
$\mu _{0}^{{C}}$	2.2114 × 10^−4^	[3]
$\gamma _{0}$	1.042 × 10^−3^	[3]
*μ*	6.25 × 10^−3^	[32]

**Table 3:** Assumption parameter value and $\Re _{0}$ value of the SIR fuzzy model for COVID-19 in Indonesia

Simulation	*ϑ*	$\Omega_{\mathrm{min}}$	$\Omega _{0}$	Ω	*θ*	*τ*	*π*	$\Re _{0}$
1	0.9	10	100	100	0	0	0	9.32
2	0.9	10	100	56	0	0	0	1
3	0.9	10	100	20	0	0	0	0.13
4	0.9	10	100	100	20%	0	0	2.05
5	0.9	10	100	100	47%	0	0	1
6	0.9	10	100	100	85%	0	0	0.58
7	0.9	10	100	100	0	2%	0	2.13
8	0.9	10	100	100	0	4.5%	0	1
9	0.9	10	100	100	0	10%	0	0.44
10	0.9	10	100	100	0	0	2%	2.13
11	0.9	10	100	100	0	0	4.5%	1
12	0.9	10	100	100	0	0	10%	0.44

Based on Table 3, it can be explained that if a treatment, vaccination, and health protocols have not been implemented, the basic reproduction number is 9.32, which means that a person with COVID-19 can infect nine other people. Meanwhile, if the effectiveness of treatment is 20% without vaccination and without implementing health protocols, it can reduce the basic reproductive number to 2.13 so that a person with COVID-19 can infect two other people. However, if the effectiveness of the treatment reaches 85%, then the basic reproduction number is reduced to 0.58, which means that the spread of COVID-19 can be controlled. Furthermore, if the effectiveness of vaccination or the effectiveness of implementing health protocols is more than 10%, then the basic reproductive number is less than 0.44, meaning that the COVID-19 outbreak will disappear in the population.

**Figure 5:**
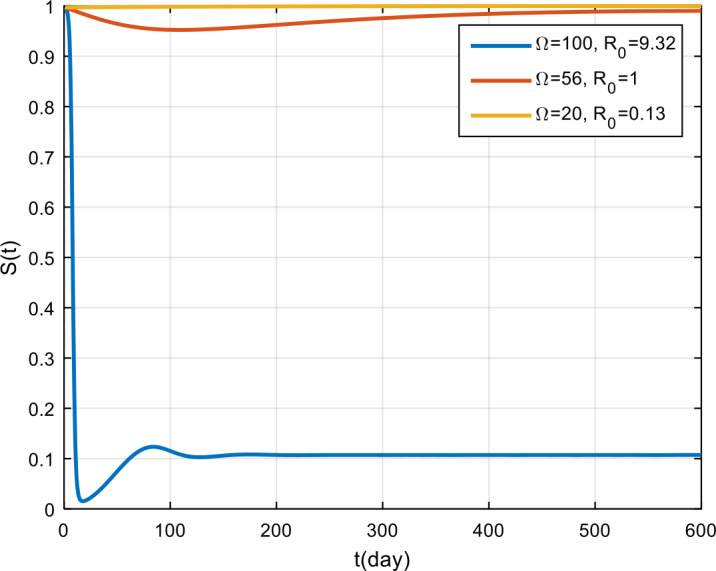
Variation in the number of suspected population for different values of Ω

**Figure 6:**
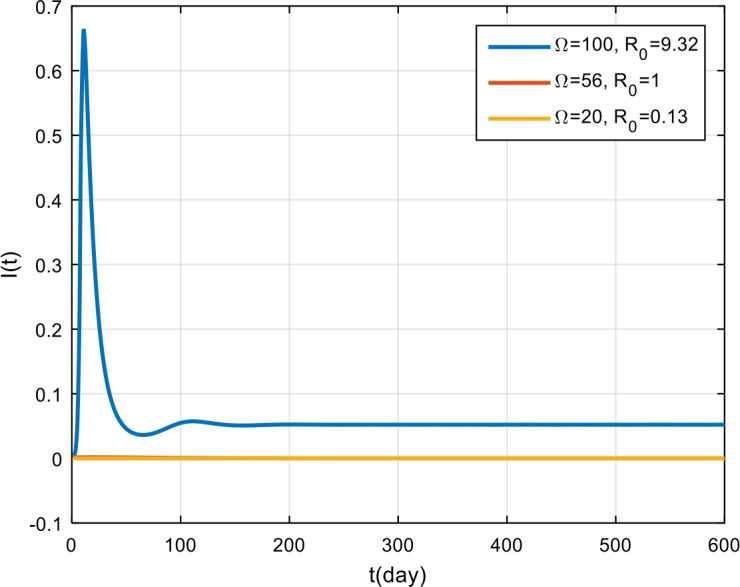
Variation in the number of infected population for different values of Ω

**Figure 7:**
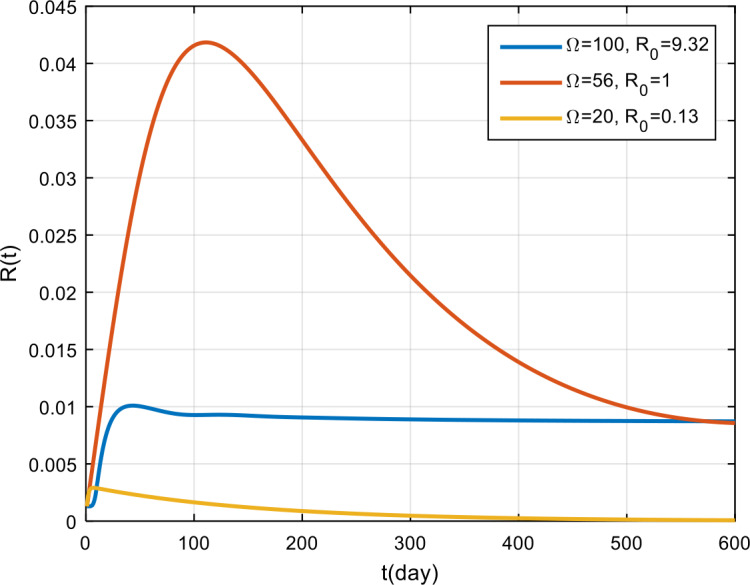
Variation in the number of recovered population for different values of Ω

The simulation results for the corona virus-load are presented in Fig. 5, Fig. 6, and Fig. 7. Based on these figures, by giving 100 the corona virus-load, the COVID-19 outbreak will never vanish in the population, and the recovery rate tends to remain, but it is smaller than those infected with COVID-19. Meanwhile, if the virus corona-load is 50, the epidemic tends to decrease, and if $\Omega = 20$, the COVID-19 outbreak will vanish in the population.

**Figure 8:**
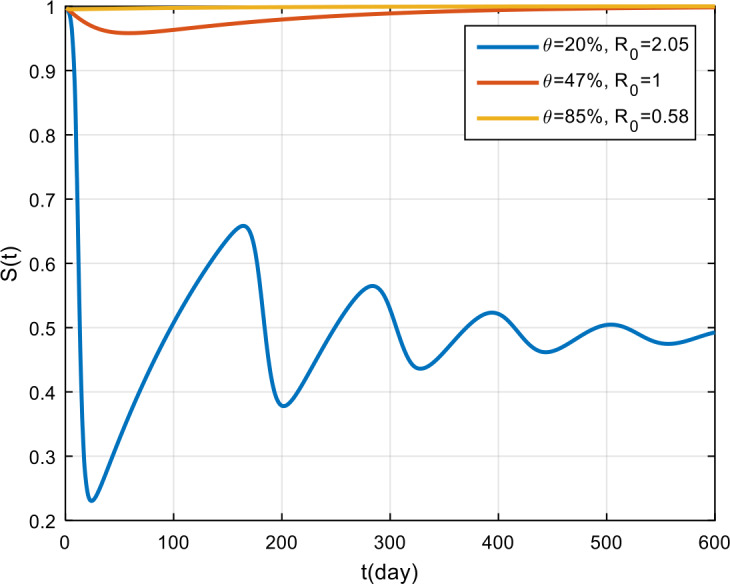
Variation in the number of suspected population for different values of *θ*

**Figure 9:**
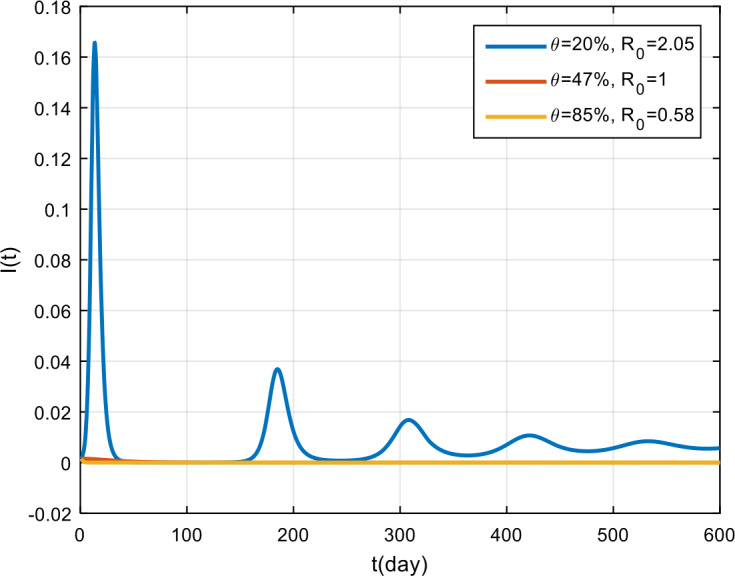
Variation in the number of infected population for different values of *θ*

**Figure 10:**
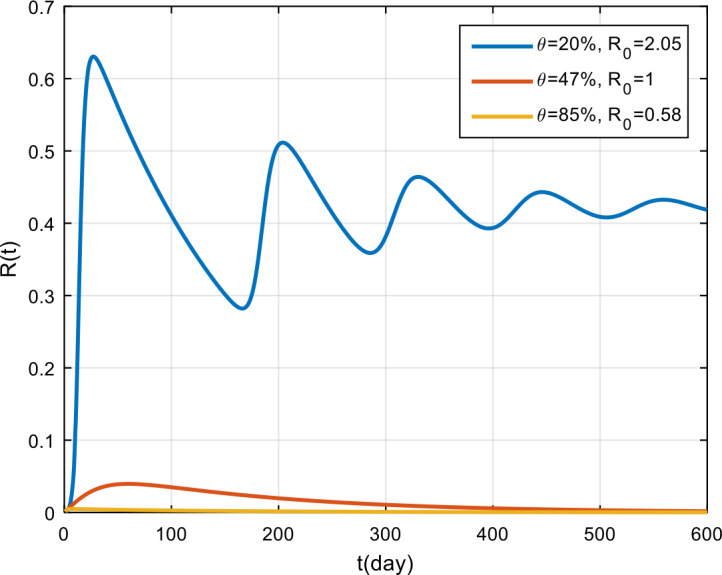
Variation in the number of recovered population for different values of *θ*

The simulation results of treatment effectiveness are presented in Fig. 8, Fig. 9, and Fig. 10. From the figures, it can be seen that if treatment is only 2% effective, COVID-19 will become endemic, while if the treatment effectiveness is 47% and 85%, the COVID-19 outbreak will disappear in the population.

**Figure 11:**
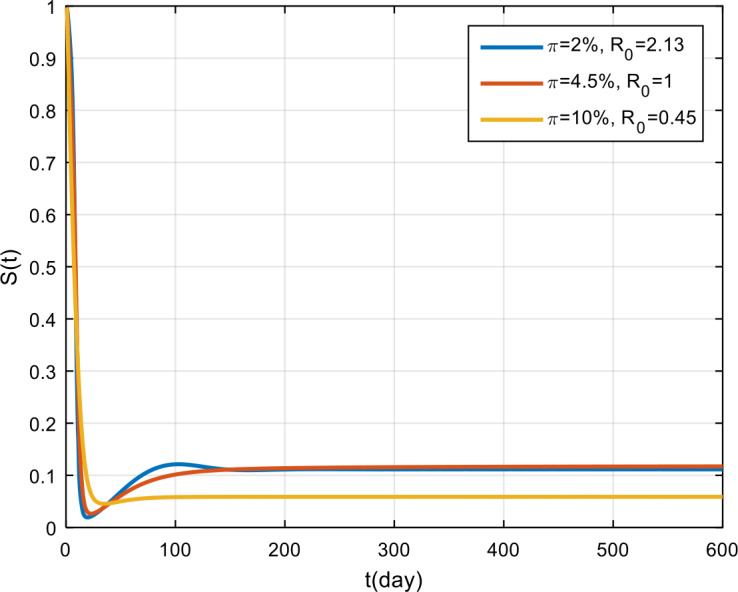
Variation in the number of suspected population for different values of *π*

**Figure 12:**
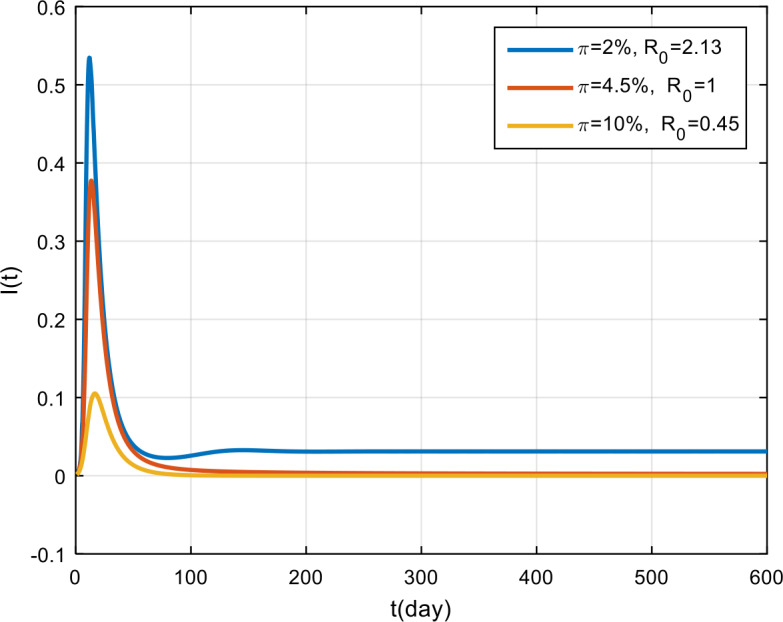
Variation in the number of infected population for different values of *π*

**Figure 13:**
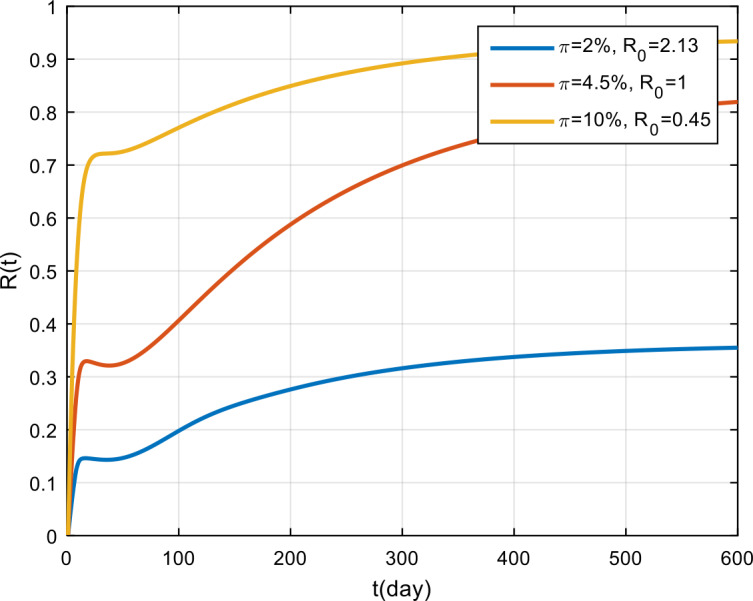
Variation in the number of recovered population for different values of *π*

The simulation results of the effectiveness of vaccination are presented in Fig. 11, Fig. 12, and Fig. 13. Based on these figures, if the effectiveness of vaccination is only 2%, then COVID-19 will become endemic, while if the effectiveness is more than 4.5%, the COVID-19 outbreak tends to vanish in the population.

**Figure 14:**
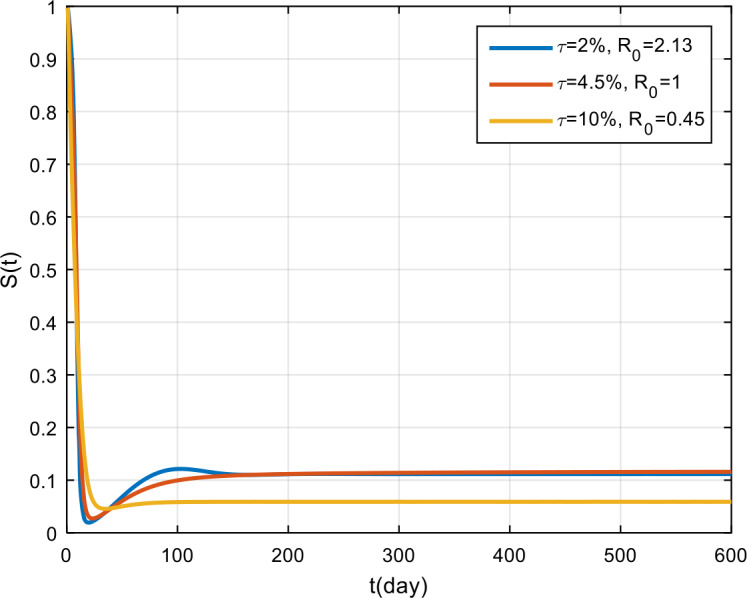
Variation in the number of suspected population for different values of *τ*

**Figure 15:**
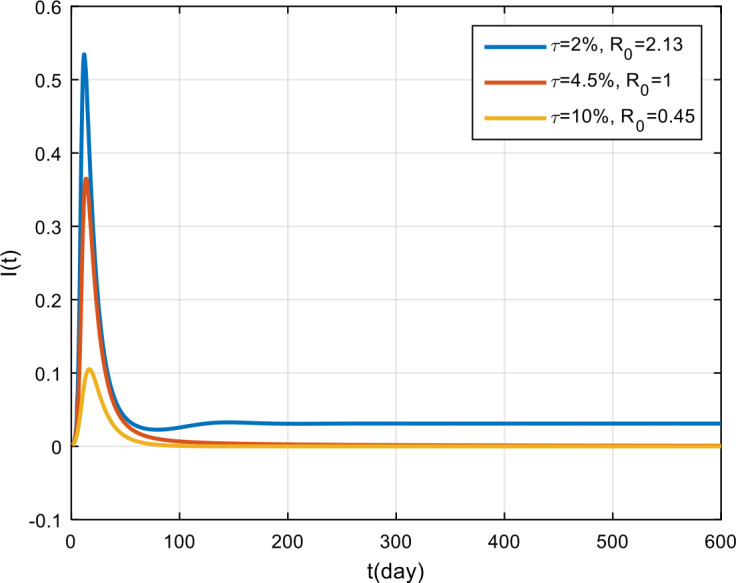
Variation in the number of infected population for different values of *τ*

**Figure 16:**
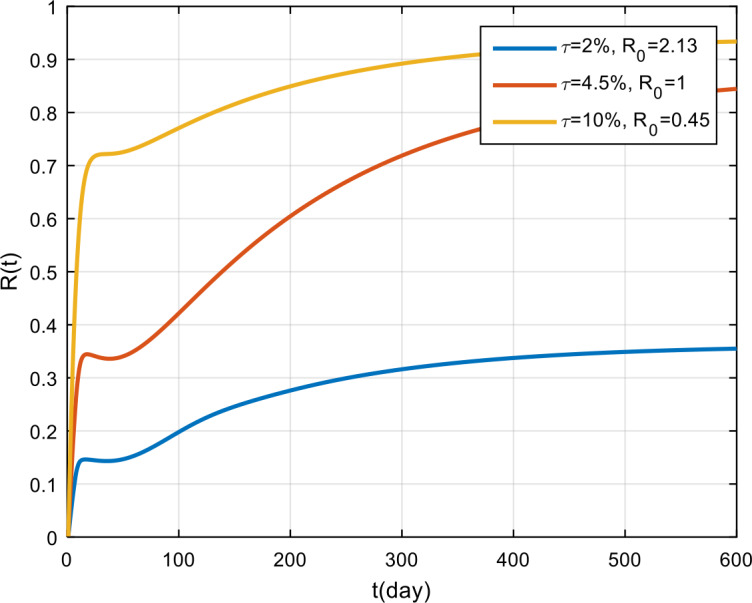
Variation in the number of recovered population for different values of *τ*

The simulation results of obedience in implementing health protocols are presented in Fig. 14, Fig. 15, and Fig. 16. Based on these figures, if obedience in implementing health protocols is only 2%, then COVID-19 will become endemic in the population. However, if obedience to follow health protocols is more than 4.5%, then the COVID-19 outbreak will vanish in the population. From the simulation results of vaccine effectiveness and the level of adherence to follow health protocols, it can be seen that, for the same *π* and *τ* values, the results will be the same. This shows that vaccination and the implementation of health protocols have the same effect on the spread of COVID-19 in Indonesia.

## Conclusion

A study using an SIR model for the spread of COVID-19 in Indonesia taking into account the factors of vaccination, treatment, implementation of health protocols and the corona virus-load has been performed. In this study, the parameters *β*, *γ*, and $\mu ^{{C}}$ are treated as membership functions of fuzzy numbers and are represented as fuzzy parameters. Those parameters depend on the corona virus load Ω. The points of disease-free equilibrium and endemic equilibrium are locally asymptotically stable for $\Re _{0}(\Omega ) < 1$ and $\Re _{0}(\Omega ) > 1$, respectively. Based on the simulation results, it is found that vaccination and the implementation of health protocols have a significant effect in slowing or stopping the spread of COVID-19 in Indonesia. Likewise, treatment has an effect in slowing or stopping the rate of infection of COVID-19 but not as much as the effect of vaccination and the implementation of health protocols.

## Data Availability

All data used are from public domains.
